# Potential of Sentinel-1 Radar Data for the Assessment of Soil and Cereal Cover Parameters

**DOI:** 10.3390/s17112617

**Published:** 2017-11-14

**Authors:** Safa Bousbih, Mehrez Zribi, Zohra Lili-Chabaane, Nicolas Baghdadi, Mohammad El Hajj, Qi Gao, Bernard Mougenot

**Affiliations:** 1 CESBIO (CNRS/UPS/IRD/CNES), 18 Avenue Edouard Belin, 31401 Toulouse CEDEX 9, France; qi.gao@isardsat.cat (Q.G.); bernard.mougenot@ird.fr (B.M.); 2 Université de Carthage/INAT/LR GREEN-TEAM, 43 Avenue Charles Nicolle, Tunis 1082, Tunisia; zohra.lilichabaane@ucar.rnu.tn; 3 IRSTEA, UMR TETIS, 500 Rue François Breton, 34093 Montpellier CEDEX 5, France; nicolas.baghdadi@teledetection.fr (N.B.); mohammad.el-hajj@teledetection.fr (M.E.H.); 4 IsardSAT, Parc Tecnològic Barcelona Activa, Carrer de Marie Curie, 8, 08042 Barcelona, Spain; 5 Observatori de l’Ebre (OE), Universitat Ramon Llull-CSIC, 08022 Barcelona, Spain

**Keywords:** Sentinel-1, radar, C-band, soil, moisture, roughness, vegetation, water cloud model, backscattering model

## Abstract

The main objective of this study is to analyze the potential use of Sentinel-1 (S1) radar data for the estimation of soil characteristics (roughness and water content) and cereal vegetation parameters (leaf area index (LAI), and vegetation height (H)) in agricultural areas. Simultaneously to several radar acquisitions made between 2015 and 2017, using S1 sensors over the Kairouan Plain (Tunisia, North Africa), ground measurements of soil roughness, soil water content, LAI and H were recorded. The NDVI (normalized difference vegetation index) index computed from Landsat optical images revealed a strong correlation with in situ measurements of LAI. The sensitivity of the S1 measurements to variations in soil moisture, which has been reported in several scientific publications, is confirmed in this study. This sensitivity decreases with increasing vegetation cover growth (NDVI), and is stronger in the VV (vertical) polarization than in the VH cross-polarization. The results also reveal a similar increase in the dynamic range of radar signals observed in the VV and VH polarizations as a function of soil roughness. The sensitivity of S1 measurements to vegetation parameters (LAI and H) in the VV polarization is also determined, showing that the radar signal strength decreases when the vegetation parameters increase. No vegetation parameter sensitivity is observed in the VH polarization, probably as a consequence of volume scattering effects.

## 1. Introduction

Soil surface and vegetation cover play a key role in various processes at the soil-vegetation-atmosphere interface, such as evapotranspiration, infiltration and runoff [1,2,3,4]. They are also essential characteristics in agricultural contexts, since they can allow improved estimations of crop conditions and requirements to be derived, and can also facilitate the optimized management of irrigation. Surface parameters have long been based on field measurements that do not allow their spatio-temporal variability to be retrieved. In this context, various studies based on optical and radar remote sensing have been proposed in the last 30 years in an effort to better characterize surface conditions and characteristics [5,6]. At present, various moisture products are proposed on a global operational basis. These are derived from microwave radiometric measurements (such as SMOS, AMSR-E) and/or scatterometer measurements (e.g., ASCAT), with a spatial resolution ranging between 10 and 40 km [7,8].

At the scale of individual agricultural fields, the monitoring of spatio-temporal variations in the vegetation’s biophysical parameters, together with the soil’s surface characteristics (water content in particular) can provide farmers with key information for the irrigation and management of their crops. Several methods have been developed to retrieve soil surface characteristics, especially surface roughness and soil moisture, from high (fine) spatial resolution synthetic aperture radar (SAR) data [9,10,11,12,13,14,15,16,17,18,19,20]. These techniques were considerably enriched by the launch of more advanced SAR sensors in the 2000s—ASAR/ENVISAT, RADARSAT, TerraSAR-X, and so forth—and make use of various types of algorithmic methods, including neural networks, look-up tables, and the inversion of physical or empirical models. 

In the case of bare soils, the radar signal scattered by the surface depends on the roughness, as well as the dielectric constant of the first centimeters of soil. The latter is directly related to the soil’s water content and the frequency of the radar sensor. The corresponding depth ranges from approximately 5 cm in the L-band to 1 cm in the X-band, in soils with a high water content [21]. Several studies have analyzed the behavior of the radar signal as a function of soil moisture content. Stronger signal sensitivity is observed at low incidence angles, between 20° and 35°, than at high incidence angles [22,23]. Different inversion approaches have been proposed, based on empirical or physical radar backscattering models (Oh model [24], IEM model [25], Dubois model [26], Baghdadi model [27], etc.). Inversion studies generally report an accuracy ranging between 3 and 6 vol. % in the case of bare soils [28,29,30,31,32,33].

Unlike the case of soil moisture, soil roughness estimations are most accurate when they are made at high angles of incidence [34], and the dynamic range of the backscattered radar signal associated with this parameter decreases as a function of increasing radar frequency [35]. The soil roughness description is based mainly on two statistical parameters: the root mean square surface height (Hrms) and the correlation length of soil height variations (L). Different approaches have been proposed to improve the roughness description [36,37,38,39,40]. Most experimental inversion studies have been restricted to the evaluation of just two or three roughness classes (smooth, medium, high) [41].

For surfaces with a vegetation cover, the backscattered radar signal depends on the characteristics of the soil and vegetation, as well as the radar configurations used for the observations. Various models have been developed in an effort to accurately retrieve soil parameters and to gain a clear understanding of the behavior of soil and vegetation components in the radar total backscattering coefficient [35]. These models are based primarily on solving the radiative transfer equation. The most commonly used model is the semi-empirical water cloud model [42], which considers the total signal to be the sum of contributions from the vegetation cover, together with a second contributor related to the soil, attenuated by its vegetation cover. Experimental studies using this model have shown that the vegetation-related terms in the model could be derived from different vegetation parameters, such as the vegetation water content or the leaf area index (LAI) [43,44]. In this context, remote sensing in the visible and infrared (i.e., “optical”) spectral ranges has demonstrated its potential for the estimation of these vegetation parameters [45,46]. Several studies have considered different satellite indices, in particular the normalized difference vegetation index (NDVI), for the estimation of the LAI of different crop types (such as wheat, grassland, rice, corn, maize, or orchards) [47,48].

When it is used in synergy with optical data, SAR imagery can thus provide valuable estimations of both soil moisture and vegetation parameters [30,44,48]. Zribi et al. [30] estimated soil moisture using ASAR images (C-band) in synergy with SPOT-5 optical images recorded over wheat plots, using the Water Cloud model, with an accuracy of approximately 0.06 m^3^/m^3^. Fieuzal et al. [48] estimated the soil moisture over irrigated wheat plots using TerraSAR-X (TSX), Radarsat-2 and Advanced Land Observing Satellite (ALOS)/Palsar data coupled with Formosat-2 and Satellite Pour l'Observation de la Terre (SPOT)-4/5 images. El Hajj et al. [44] combined X-band data retrieved from TSX with data derived from CosmoSky-Med (CSK) sensors, as well as NDVI, LAI, FAPAR (Fraction of Absorbed Photosynthetically Active Radiation) and FCOVER (Fraction of Cover) products from SPOT-4, SPOT-5, LANDSAT-7 and LANDSAT-8 images, using a water cloud model to obtain soil moisture estimations over grassland areas.

Despite the considerable contributions of optical data to the estimation of biophysical vegetation parameters, due to saturation effects, their application to the case of high or very dense vegetation (with high values of LAI) is often inadequate. Secondly, in the case of regions with a high average level of cloud cover, such as tropical zones, high quality optical acquisitions are very limited and/or infrequently recorded. In this context, several studies have also assessed the sensitivity of SAR signals at different radar wavelengths (mainly the L, C and X-bands) to vegetation conditions [49,50,51,52]. For example, Fontanelli et al. [52] revealed the high sensitivity of TSX and CSK data to increasing LAI in the case of cereal cover (~0.6 dB/1 m^2^/m^2^), with a coefficient of determination greater than 0.70. El Hajj et al. [44] also reported the high sensitivity of X-band data to LAI, when the soil moisture lies between 10 and 20 vol. % over grassland areas. Baghdadi et al. [51] analyzed the potential interpretation of TSX data as a function of sugarcane height, showing that the backscattering coefficient increases with crop height at a high incidence angle (47°).

The Sentinel-1 constellation was launched in 2014 (Sentinel-1A) and 2016 (Sentinel-1B), with both satellites carrying a C-band radar payload. Thanks to its short temporal repeat frequency (six days), as well as the high spatial resolution (10 m) provided by its payload, this constellation is making it possible to achieve regular operational estimations of the conditions and characteristics of the continental surface. 

In this context, the main objective of the present study is to make use of a large database to analyze the potential of Sentinel-1 (S1) data for the assessment of different soil and cereal parameters. The synergetic use of S1 radar data with Landsat high-resolution optical data is also analyzed. Section 2 describes the study site, satellite images and ground measurement database. Section 3 provides a statistical analysis of the relationships established between the backscattered radar signal and the surface parameters (soil and vegetation). Section 4 discusses the performance of the different backscattering models for bare and covered soils. Our conclusions are provided in Section 5.

## 2. Study Site and Database

### 2.1. Description of the Study Site

**Figure 1:**
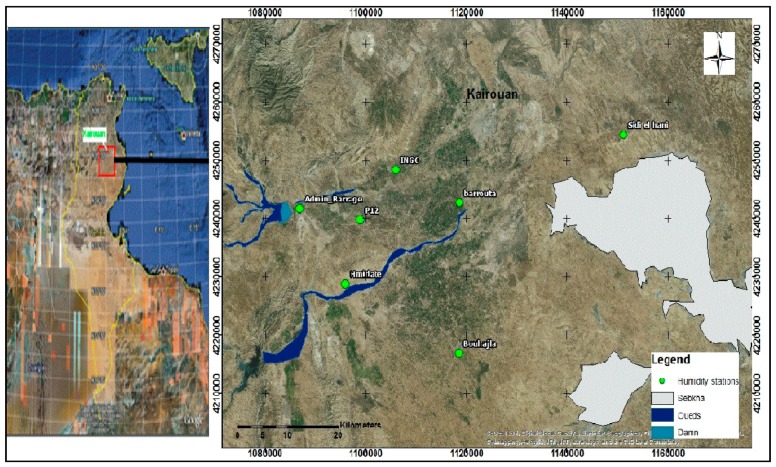
Location of the study site in Tunisia.

The study site is located on the Kairouan plain (9°23′–10°17′ E, 35°1′–35°55′ N; Figure 1), in Central Tunisia [30]. This region is exposed to a semi-arid Mediterranean climate, characterized by a dry summer season, with precipitation occurring mainly in autumn and spring, leading to an annual average rainfall of approximately 300 mm. This precipitation is highly variable in time and space, with frequently occurring drought events [30]. The average annual temperature is 19.2 °C, with the minimum occurring in January and the maximum in August. The average potential evapotranspiration is close to 1600 mm. The landscape in this area is mainly flat and the vegetation is dominated by agricultural plants (cereals, olive trees, and market gardens). Various different cereal crops are grown, and their rotation is typical of semi-arid regions. The Kairouan plain aquifer is the largest in central Tunisia. Surface and groundwater streams are drained into the Sebkha Kelbia, a large salt lake.

### 2.2. Database

#### 2.2.1. Satellite Data

(a) Sentinel-1 radar data

The first S-1A satellite was launched on 3 April 2014 and was followed by the S-1B Sentinel satellite on 25 April 2016. This dual-satellite constellation offers a six-day repeat frequency for all regions of the globe. The SAR payloads use a C-band frequency of 5.4 GHz and have the following standard operating modes: Strip Map (SM); Interferometric Wide Swath (IW); Extra Wide Swath (EW), and; WaVe (WV). In the present study, 10 IW S1 images were analyzed. They were characterized by a 10 × 10 m spatial resolution, dual VV and VH polarization measurements, and an incidence angle of approximately 39–40° (Table 2). These were acquired between December 2015 and March 2017. All of the images were generated from the high-resolution, Level-1 Ground Range Detected (GRD) product. The data were preprocessed using the Sentinel-1 Toolbox, in four steps:

- Thermal noise removal;
- Radiometric calibration;
- Terrain correction using Shuttle Radar Topography Mission (SRTM) Digital Elevation Model (DEM) at 30 m;
- Filtering of speckle using a Lee filter.

The calibration is designed to convert the digital values of the raw images into backscattering coefficients (*σ*^0^).

(b) Landsat optical data

Seven Landsat-8 images downloaded from the USGS website (http://earthexplorer.usgs.gov/) were used to provide optical wavelength observations of the study area. The atmospheric correction of these images was performed using NASA’s Landsat Ecosystem Disturbance Adaptive Processing System (LEDAPS). This software uses the 6S (Second Simulation of a Satellite Signal in the Solar Spectrum) radiative transfer model to estimate the surface reflectance. The NDVI vegetation index, expressed by NDVI = (RNIR − RRED)/(RNIR + RRED), where RNIR is the near-infrared (NIR) reflectance and RRED is the red reflectance, was calculated from the optical images. The data used in this analysis were derived from images recorded in the absence of clouds.

#### 2.2.2. Ground Measurements

**Figure 2:**
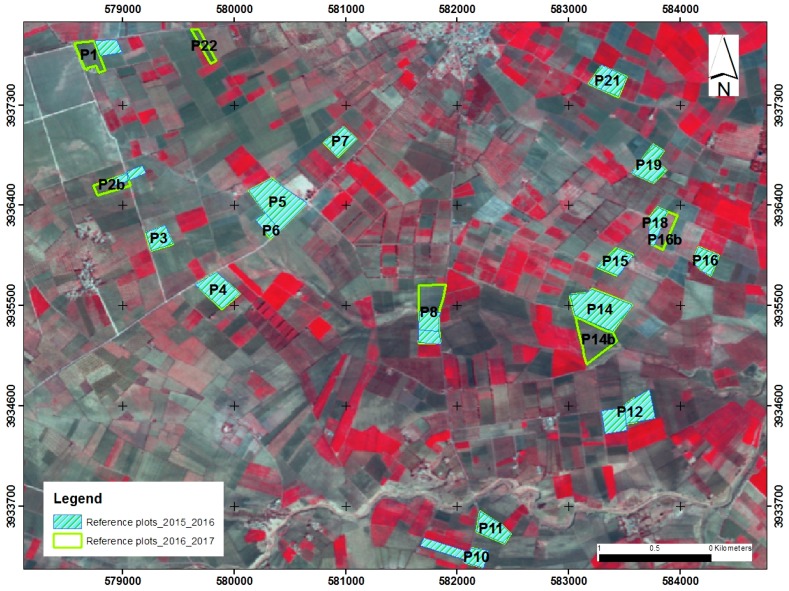
Location of reference fields on the study site (Landsat image).

During two cycles of cereal fields (from December 2015 to May 2016, and from December 2016 to March 2017), ground measurements were carried out at the same time as several S1 sensor acquisitions (Figure 2). In the Kairouan plain, early December generally corresponds to the sowing season, whereas the end of March corresponds to the period of maximum cereal growth. Cereal senescence occurs prior to harvesting, between mid-April and the end of May. For each cycle, more than 20 reference fields ranging in size between 2 and 5 ha were considered, under conditions of either bare soil with varying degrees of roughness, or bearing cereals at different stages of growth (irrigated and non-irrigated) (Table 1). The first campaign in each agricultural cycle (during the month of December) was carried out on bare soil, prior to cereal growth. The fields are sufficiently large to ensure that the mean values of the radar measurements, which are affected by the presence of speckle noise, are sufficiently accurate.

They were observed during both cereal cycles; moisture and roughness measurements were carried out for the soil characterization. Leaf area index (LAI) and canopy height (H) measurements were recorded for the fields with a vegetation cover. Table 2 lists the dates of the field campaigns, and the corresponding types of measurement.

(a) Soil Moisture

On each of the dates listed in Table 2, approximately 20 handheld Theta Probe measurements were made in each reference field (irrigated or non-irrigated) at a depth of 5 cm. The samples were taken from various locations in each reference field, within a two-hour time frame of the concurrent satellite acquisitions, and the Theta Probe measurements were calibrated with gravimetric measurements recorded during previous campaigns [30]. This semi-arid region is characterized by strong temporal variations in rainfall, which were revealed by the very high range of soil moisture content values (between 3.9 vol. % and 45 vol. %) measured during the experimental campaigns (Table 2). 

**Figure 3:**
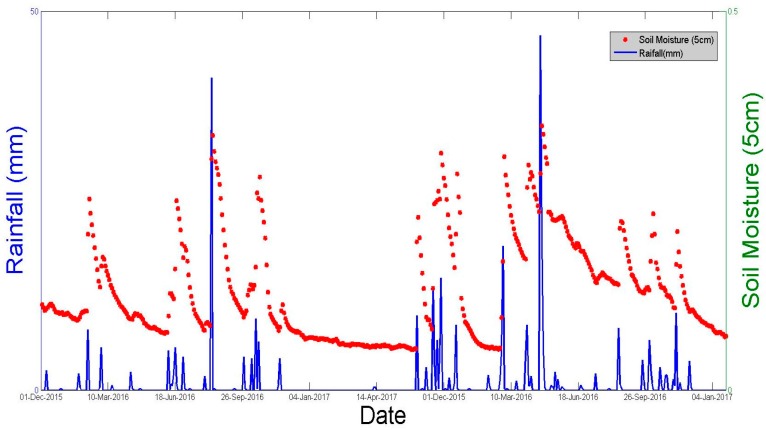
Daily precipitation and mean surface moisture over the studied site during the measurement campaigns.

In addition to the moisture measurements carried out in reference fields, a network of seven continuous Theta Probe stations (Figure 1), installed at various bare soil locations over the studied site, was used to provide moisture measurements every 3 h. At each station, the measurements were made at depths of 5 and 40 cm. Figure 3 plots the daily values of precipitation and the mean daily volumetric surface soil moisture, estimated by calculating the mean value of the continuous recordings given by Theta Probe measurements at a depth of 5 cm during the two studied agricultural seasons. A strong correlation can be noted between the soil moisture estimations and the precipitation events.

(b) Soil roughness parameters

Radar signals are highly sensitive to the geometry of scattering surfaces. In the present context, roughness is a measure of the variations in micro-topographic height of the soil surface. Measurements were made using a 1 m long pin profiler, with a separation of 2 cm between successive needles. For each test plot, five parallel and five perpendicular profiles were compared in order to take the possible effects of directional roughness retrieval into account. The roughness profiles are considered to be stationary and ergodic. Two statistical parameters are then estimated: the root mean square surface height (Hrms) and the correlation length (L) [34]. These were computed from the correlation functions of the heights of the surface profiles. We considered two roughness measurement campaigns during each agricultural year, which was sufficient to account for most variations in soil roughness (Table 2). The soil roughness can vary over a wide range of values, mainly in autumn, prior to the sowing of cereals. After sowing, the soil is no longer tilled and is affected only by very small temporal variations in roughness. During all measurement campaigns, Hrms ranged between 0.56 and 4.55 cm, and the correlation length varied between 1.96 and 13.14 cm.

(c) Vegetation parameters

Vegetation cover measurements are carried out to characterize several parameters: the leaf area index (LAI) and canopy height (H), as described in the following.

**Leaf Area Index (LAI)**

The LAI is defined as the total one-sided area of leaf tissue per unit ground surface area. According to this definition, the LAI is a dimensionless quantity. For each test field, 20 hemispherical digital images were used. These were processed by analyzing the canopy gap fraction, in order to retrieve this vegetation parameter [53]. The measurements were applied four times during each agricultural season. During all measurement campaigns, the computed value of LAI ranged between 0 and 5 (Table 2). The highest values of LAI were observed mainly in irrigated test fields, between the end of March and the beginning of April. 

**Vegetation Height (H)**

Approximately 20 vegetation height measurements were considered for each reference field and for each date. These were averaged, to retrieve a mean height value for each test field. In the case of H, measurements were carried out four times during each agricultural season. The tallest vegetation heights lie in the range (90–110 cm) and are reached towards the end of March or the beginning of April (Table 2).

**Land use**

**Figure 4:**
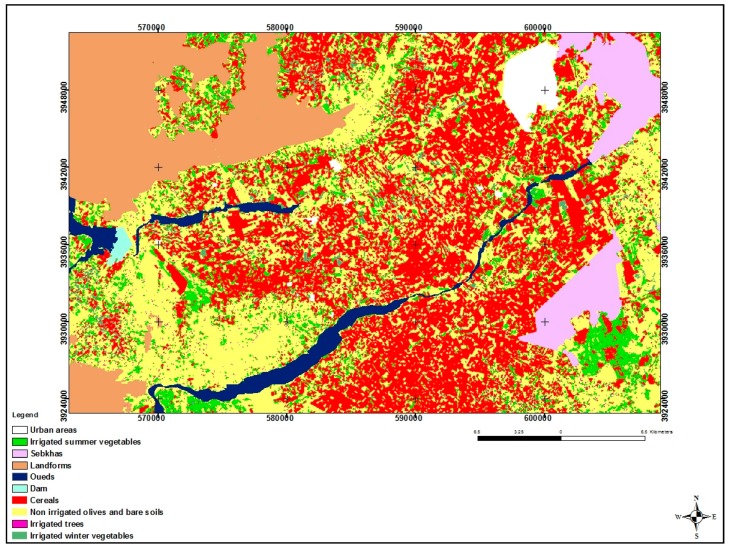
Land use map.

Land use maps were produced in 2017. This process relies on the decision-tree method proposed in [30] and was applied to four LANDSAT images (recorded in late autumn, early winter, spring and summer). Ten classes of land-use classification were identified: non-irrigated olive trees; irrigated trees; irrigated winter vegetables; irrigated summer vegetables; cereals; urban areas; sebkha areas; landforms; dams, and; oueds. These remotely sensed classifications were validated during a ground verification campaign, involving more than 100 fields with various types of land use. The results reveal an identification accuracy of approximately 75%. Figure 4 shows the Resulting Land Use Map for the 2016–2017 season.

## 3. Results and Discussions 

### 3.1. Relationships between NDVI and Vegetation Parameters

**Figure 5:**
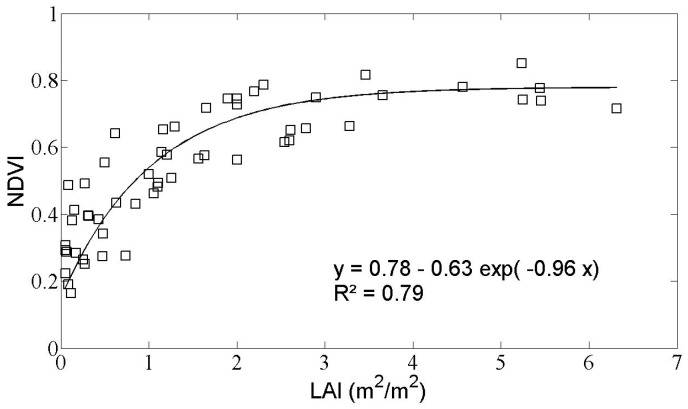
Relationship between LAI ground measurements and normalized difference vegetation index (NDVI) derived from optical images.

The values of NDVI corresponding to each pixel were obtained from optical Landsat images, and then averaged for each reference field. Figure 5 provides a plot of the NDVI as a function of the LAI ground measurements, showing that the NDVI saturates at a value of 0.78 when the LAI reaches a value of around 3 m^2^/m^2^.

An exponential function has been proposed to describe this relationship [30,44,47]:

(1) $$NDVI=NDVI_{\max}+(NDVI_{soil}-NDVI_{\max})\times e^{-k\;LAI}$$

where NDVI_max_ (0.78) is the NDVI corresponding to the maximum value of LAI, NDVI_min_ is the bare soil NDVI (=0.15), and *k* is the extinction coefficient (=0.96). The coefficient of determination R^2^ for this data is 0.79, with a RMSE (relative mean square error) of 0.96 m^2^/m^2^. Courault et al. [47] reported a value of *k* = 0.71, derived from Formosat-2 images acquired over a larger area in Southeast France including wheat and rice fields, as well as irrigated grasslands. In the case of Landsat images of grassland plots in France, El Hajj et al. [44] reported a value of *k* = 0.69.

### 3.2. Radar Signal Analysis

#### 3.2.1. Relationship between Radar Signal and Soil Moisture

**Figure 6:**
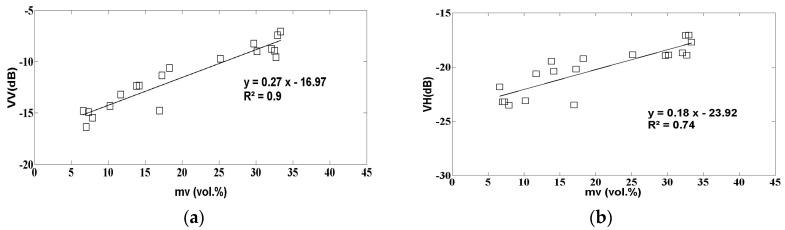

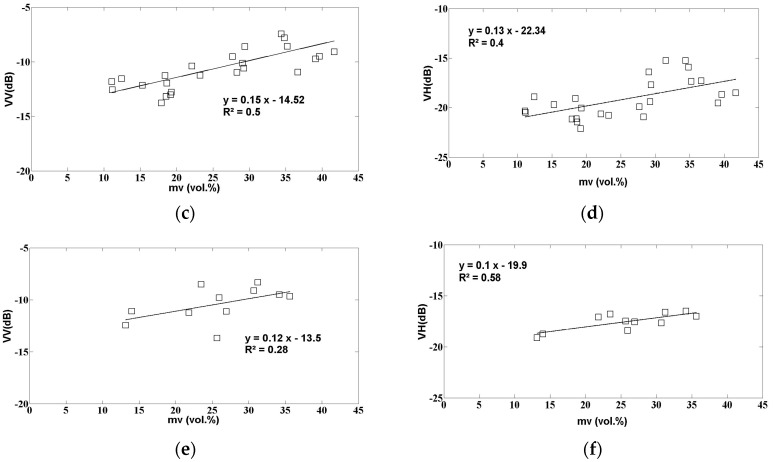
S1 radar data as a function of soil moisture: (**a**) VV polarization, NDVI < 0.25; (**b**) VH polarization; NDVI < 0.25; (**c**) VV polarization, 0.25 < NDVI < 0.5; (**d**) VH polarization, 0.25 < NDVI < 0.5; (**e**) VV polarization, NDVI > 0.5; (**f**) VH polarization, NDVI > 0.5.

In order to minimize the influence of vegetation heterogeneity, the sensitivity of the radar signals to soil moisture was analyzed for three classes of NDVI. The first of these is defined by NDVI < 0.25, corresponding (in general) to bare or poorly covered soils. The second class is defined for 0.25 < NDVI < 0.5, corresponding to a medium vegetation density, and the third class is defined for high NDVI values (NDVI > 0.5). The NDVI threshold defined at 0.5 corresponds to an LAI equal to ~1. Figure 6 plots the VV and VH radar signals as a function of soil moisture for the three NDVI classes. The sensitivity of radar signals to soil moisture can be seen to decrease with increasing NDVI. The vegetation component, which attenuates the signal scattered by the soil, thus influences the radar sensitivity to variations in soil moisture. As an example, for the VV polarization the sensitivity decreases from 0.27 dB/vol. % for the weak NDVI values (NDVI < 0.25), and to 0.12 dB/vol. % for the highest NDVI values (NDVI > 0.5). The signal strength sensitivity is clearly higher in the VV than in the VH polarization, for all three classes of NDVI. As an example, in the first NDVI class the sensitivity is 0.27 dB/vol. % for VV and 0.18 dB/vol. % for VH. In the VH polarization, the lower soil moisture sensitivity can be explained by the higher sensitivity of the radar signals to the volume component of medium scattering [35]. For the two highest NDVI classes, the correlation between moisture and radar signals is the lowest, due to the disruptive effect of increasing vegetation cover and the absence of field data at low values of soil moisture.

The behaviors found for S-1 are generally consistent with those reported in previous studies, in the case of data acquired in the C-band [30,54].

#### 3.2.2. Relationship between Radar Signal and Soil Roughness

**Figure 7:**
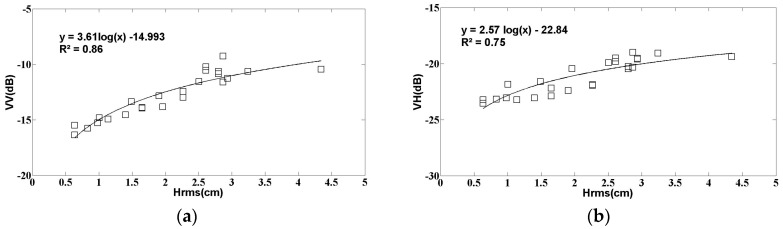

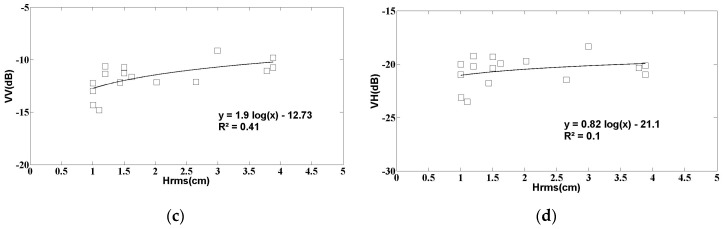
S1 radar data as a function of the Hrms roughness parameter in two soil moisture (mv) classes: (**a**) VV polarization, mv ≤ 10 vol. %; (**b**) VH polarization, mv < 10 vol. %; (**c**) VV polarization, 10 vol. % < mv < 20 vol. %; (**d**) VH polarization, 10 vol. % < mv < 20 vol. %.

**Figure 8:**
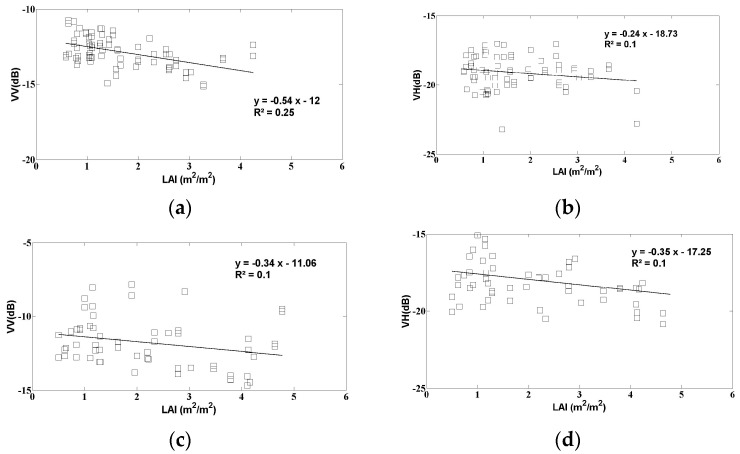
S1 data as a function of LAI for two classes of soil moisture (mv): (**a**) VV polarization, mv ≤ 15 vol. %; (**b**) VH polarization, mv ≤ 15 vol. %; (**c**) VV polarization, mv > 15 vol. %; (**d**) VH polarization, mv > 15 vol. %.

As noted in the introduction, in the case of bare soils, the soil roughness has a very strong influence on the behavior of radar signals. In the present study, the relationship between radar signals and the parameter Hrms was analyzed. As shown in several preceding studies [16,34], it is valid, to a good approximation, to take the linear sum of the radar signal contributions produced by roughness and soil moisture. In order to avoid the influence of moisture heterogeneities, the relationship between roughness and radar signals is analyzed for two moisture classes: a class with low moisture values ≤ 10 vol. % and a second class with soil moisture (mv) ranging between 10 vol. % and 20 vol. %. In the database used for the present study, since a very small number of bare soil measurements (8) were found to correspond to the highest moisture values (>20 vol. %), an additional third class, with mv > 20 vol. %, was not considered. Figure 8 shows the relationship between the soil roughness parameter (Hrms) and the S1 signals in the VV and VH polarizations, for each of these two moisture classes. A high correlation, greater than 0.7 in the case of low moisture values (mv < 10 vol. %), is obtained between the radar signal strengths and the roughness parameters. In this moisture class, the VV and VH data have approximately the same behavior, characterized by roughness-induced signal strength variations of approximately 6 dB. It can be seen in Figure 7 that in the high moisture class (10% < mv < 20%) there is just a small number of data points (16 observations), with no reference fields having a value of Hrms smaller than 1 cm. This leads to a lower correlation between roughness and radar signal.

#### 3.2.3. Relationship between Radar Signal and Cereal Parameters

In this section, the sensitivity of radar signals is discussed, for the case of two vegetation parameters: LAI and H. Figure 8 shows the relationship between the radar signals (VV and VH polarizations) and the LAI, for two moisture classes: mv ≤ 15 vol. % and mv > 15 vol. %. Just two moisture classes were proposed, in order to include a sufficiently large number of observations. In the VV polarization, the radar sensitivity to LAI leads to a dynamic range of approximately 2 dB in both moisture classes, with the signal decreasing with increasing LAI. This behavior is due to the fact that when the LAI increases, it leads to stronger vegetation-induced attenuation of the backscattered soil signals.

The behavior of the VH radar signals as a function of LAI is different to that observed in the VV polarization, and vegetation cover appears to have a relatively weak influence on the radar signal strength. In practice, when the vegetation-induced attenuation increases, this is accompanied by a decrease in the soil component of the total radar signal, and vegetation scattering (in particular, volume scattering, which is stronger in the VH polarization) increases with increasing LAI. Fontanelli et al. [52] have shown similar results for HH and VV-polarized radar observations (TSX data), in which the signal strength decreases as a function of increasing LAI. However, radar signals are not affected in the same way by all types of vegetation cover, since the radar signal is strongly dependent on the geometric structure characterizing each type of vegetation. As an example, El-Hajj et al. [44] observed a positive relationship between signal strength and LAI in the case of grass-covered fields.

**Figure 9:**
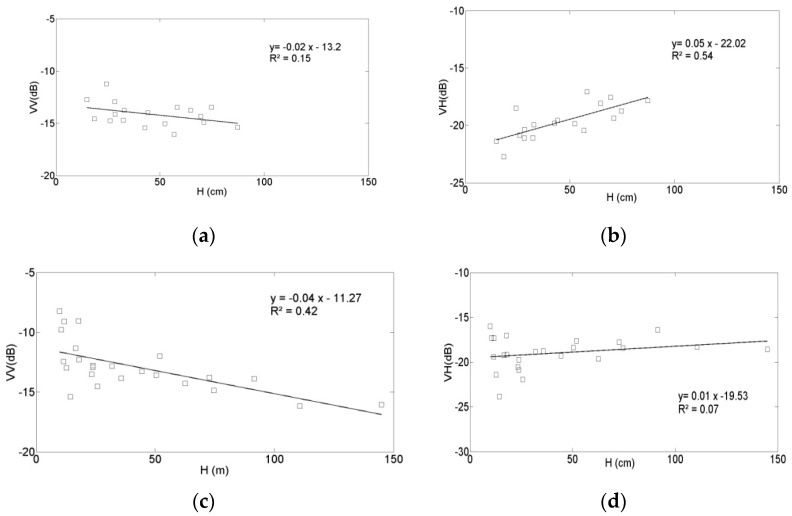
Sentinel-1 (S1) radar data as a function of H in two soil moisture (mv) classes: (**a**) VV polarization, mv < 15 vol. %; (**b**) VH polarization, mv < 15 vol. %; (**c**) VV polarization, mv > 15 vol. %; (**d**) VH polarization, mv > 15 vol. %.

Figure 9 plots the VV and VH signals as a function of the second vegetation parameter, the vegetation height (H). This analysis was made for vegetation heights recorded until the beginning of April, corresponding to the end of the local period of plant growth. After this date, although the height of the plants remains constant, the vegetation is characterized by a decreasing level of vegetation water content. As in the case of the LAI observations, two moisture classes, mv ≤ 15 vol. % and mv > 15 vol. %, were considered. In the VV polarization, very similar behaviors are observed, as in the case of the LAI parameterization. The VV radar signal strength can be seen to decrease with increasing vegetation height. In the low moisture class, there are no data points corresponding to vegetation higher than 100 cm, which could explain the observed decrease in correlation with respect to the values determined for the high moisture class. As shown in Figure 9, in the VH polarization the radar signal strength increases with increasing vegetation height. This behavior appears to be contradictory to that observed in Figure 8, which reveals a weakly decreasing VH signal strength as a function of increasing LAI. This apparent discrepancy can be explained by the fact that ground-truth measurements began when the vegetation height was approximately 10–15 cm, following the first LAI campaigns. This means that the influence of the soil was lower, and that volume scattering from the vegetation (Figure 9) could have dominated the backscattered radar signals.

## 4. Simulation of S1 Data with Backscattering Models

In this section, two models are used to simulate radar backscattering over fields characterized by two different types of land use: bare soil and soil covered by cereals. In the case of bare soils, an empirical model was used. This takes the influence of moisture and roughness into account. In the case of surfaces with a vegetation cover, the water cloud model was applied [42]. This is particularly useful for the inversion of radar data over agricultural soils [30].

### 4.1. Bare Soil Backscattering Model

In the present study, two empirical expressions were used to model the radar backscattering over bare soil, for the VV and VH polarizations. These can be written as:

(2) $$\sigma_{vv}^{soil}=\alpha_{vv}\;mv+\beta_{vv}\;\log(Hrms)+\delta_{vv}$$

(3) $$\sigma_{vh}^{soil}=\alpha_{vh}\;mv+\beta_{vh}\;\log(Hrms)+\delta_{vh}$$

The observations describing the bare soil reference fields are separated into two datasets. The first of these includes 45 fields and is used for the calibration of the proposed expression, whereas the second set of (45) fields is used to validate the results. Table 3 lists the calibrated model parameters for the VV and VH polarizations and corresponding statistical parameters (RMSE and R^2^). The two datasets correspond to multi-temporal measurements made in two sets of distinctly different reference fields.

**Figure 10:**
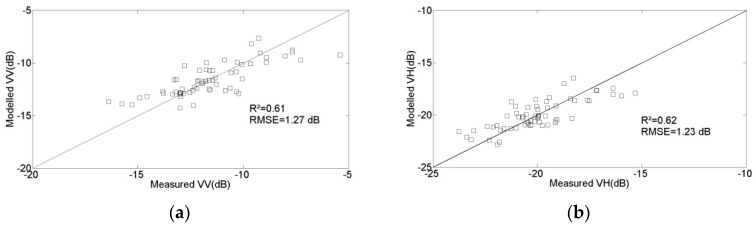
Empirical model validation: (**a**) VV polarization; (**b**) VH polarization.

Figure 10 plots the results obtained through validation of the proposed model, by comparing the signal strengths predicted by the model with the real data derived from the bare soil surfaces described in the second dataset. This figure shows that there is a strong agreement between the simulations and measurements, with an RMSE equal to 1.27 and 1.23 dB for the VV and VH data, respectively, and a bias equal to 0.1 and 0.2 dB for the VV and VH data, respectively. These results highlight the potential of this model for the analysis and inversion of radar data over bare soils.

### 4.2. Validation of the Water Cloud Model

The water cloud model developed by Attema and Ulaby [42] was used to model the radar signal backscattered by covered surfaces. At an incidence angle *θ*, the backscattering coefficient in this model is given by the following expression:

(4) $$\sigma^{0}=\sigma_{canopy}^{0}+\sigma_{canopy+soil}^{0}+\tau^{2}\sigma_{soil}^{0}$$

where *τ*^2^ is the two-way vegetation transmissivity. The first term represents scattering due to the vegetation, whereas the second term is related to multiple scattering effects, and the third term represents soil scattering attenuated by the vegetation cover. The second term, which can be neglected in the case of wheat scattering, is used to account for double-bounce scattering, the effects of which are stronger in the VV than in the VH polarization. Expression (4) can thus be simplified to:

(5) $$\sigma^{0}=\sigma_{canopy}^{0}+\tau^{2}\sigma_{soil}^{0}$$

With

(6) $$\tau^{2}=\exp(-2\;B\;V1\;\sec\theta )$$

And

(7) $$\sigma_{canopy}^{0}=A\;V1\;\cos\theta \;\left(1-\tau^{2}\right)$$

where V1 is a parameter related to the vegetation. In the present case we consider the NDVI, which is strongly related to the vegetation parameters. The parameters A and B depend on the type of canopy. This formulation represents a first-order solution for the radiative transfer equation through a weak medium, where multiple scattering is neglected. As in the case of bare soils, we consider two datasets, with the first being used for the calibration of A and B, and the second for validation of the retrieved model. 

The empirical model proposed in expression (2) was used to represent the soil contribution.

In the present study, two different data sets were used, with different soil and vegetation conditions (moisture, roughness, vegetation index (NDVI)). The first of these contains data recorded from 110 reference fields, and is used to determine the parameters A and B, which are then used to calibrate the model. The second dataset describes a different set of 110 reference fields, which are used to validate the calibrated model. As the results described in Section 3.2 reveal a very low sensitivity of VH signals to vegetation cover, which is probably due to a volume scattering effect, in the water cloud model we consider the VV polarization only. The calibration process allows the empirical parameters A and B to be determined as: A = 0.06; B = 0.42.

**Figure 11:**
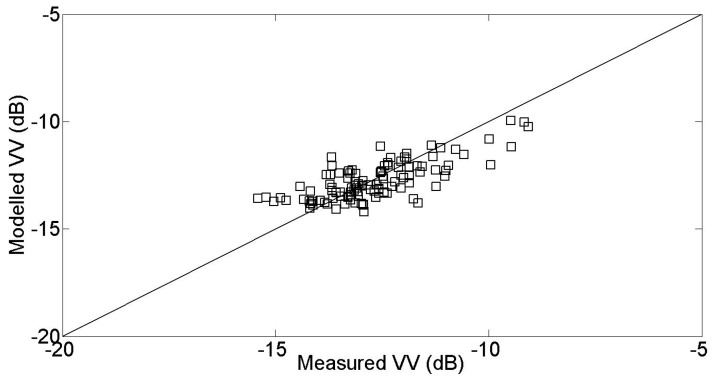
Water cloud model validation for the VV polarization.

In Figure 11, the modeled VV radar strength is compared with the measured values taken from the second validation dataset. These are based on measurements made over reference fields, which are different to those used for the calibration. The modeled signal strengths can be seen to be in very good agreement with the real radar signal. This agreement was observed for a large range of vegetation types (NDVI between 0.14 and 0.69), soil moistures (ranging from 6.4 vol. % to 37.1 vol. %) and soil roughness values (ranging between 0.94 cm and 2.02 cm). The RMSE is equal to 0.84 dB and the bias is equal to 0.08 dB. 

## 5. Conclusions

In this study, the potential use of Sentinel-1 radar signals for the assessment of different surface states (soil moisture and soil roughness, vegetation cover parameters) is analyzed. The results are based on the use of a large database, describing two agricultural years of cereal production (2015–2016 and 2016–2017). Remotely sensed data recorded by the Sentinel-1 and Landsat payloads are compared with in situ measurements, corresponding to more than 20 reference plots, which are characterized either by bare soil, or by cereal coverage. This study has made it possible to determine the sensitivity of Sentinel-1 signals to soil moisture. The radar sensitivity is clearly determined, through the interpretation of data corresponding to three classes of vegetation, ranging from bare soil to dense canopies. The radar signal sensitivity is found to decrease with increasing NDVI index, and to be higher in the VV polarization than in the VH polarization. This suggests that VV data have a greater potential for the estimation of soil moisture. Concerning the soil roughness, the Sentinel-1 VV and VH radar signals are found to be highly sensitive to the roughness parameter Hrms, with a dynamic range reaching 6 dB for both polarizations. A correlation greater than 0.7 is determined for an empirical logarithmic relationship between the radar signals and Hrms, for the case of plots in the low moisture class (mv ≤ 10 vol. %). Two classes of moisture (mv ≤ 15 vol. %, mv > 15 vol. %) are used to analyze the role of vegetation cover, revealing that the VV radar signals are highly sensitive to the LAI. The dynamic range of the radar signals is equal to 2 dB and 1.5 dB, for these two classes of soil moisture. There is almost no significant correlation between signal strength and soil moisture in the case of the VH polarization. 

The radar signals expected over bare soils are modeled using a simple empirical expression, which includes a linear relationship with respect to soil moisture, and a logarithmic behavior with respect to roughness. When the calibrated model is validated with real data recorded over a variety of reference fields, it is found to be very accurate: the RMSE is equal to 1.27 and 1.23 dB, for the VV and VH polarizations, respectively. 

In the case of radar signals observed over covered soils, a calibrated water cloud model is proposed, using the NDVI as a parameter to describe the vegetation. This model is proposed for the VV polarization only. As in the case of bare soils, when the model is validated with a set of reference fields, the simulated signal strengths are found to be in good agreement with the real measurements (RMSE equal to 0.84 dB). Despite its simplicity, the water cloud model is able to correctly describe the behavior of radar signals, as a function of soil moisture and vegetation parameters. The empirical calibration parameters derived from this analysis have been adapted to the case of agricultural plots with a cereal cover. In future studies, they will be tested at a different site, characterized by different types of vegetation cover.

This study reveals the high potential of Sentinel-1 data, when combined in synergy with optical (Landsat) images, for the recovery of moisture and vegetation characteristics. It is clearly shown that VV polarized signals have a greater sensitivity to these surface parameters, and that remotely sensed Sentinel-1 radar data can be inverted and used for the mapping of soil and cereal cover characteristics.

## Figures and Tables

**Table 1:** Land cover during 2015–2017.

Land Cover	2015–2016	2016–2017
Bare soil	P2, P4, P5, P6, P8a, P10, P18, P21, P21b	P5, P12, P18
Pasture	P2b, P9, P15, P22	P10, P15
Irrigated wheat	P1, P3, P7, P8b, P11, P12, P13, P16, P19	P1, P3, P7, P8, P11, P14b, P16, P16b, P19, P22
Rain-fed wheat	P14	P2b, P4, P6, P14, P21

**Table 2:** Radar and ground measurement campaigns during 2015–2017. Radar image configurations; Root mean square surface height (Hrms); leaf area index (LAI); vegetation height (H).

Date	Radar Image (Polarisation, Angle)	Soil Moisture (vol. %)	Hrms (cm)	LAI (m^2^/m^2^)	H (cm)
6 December 2015	VV/VH, 39–40°	[5.2–26.49]	[0.56–2.93]		
3 February 2016	VV/VH, 39–40°	[5.29–25]	-	[0.64–4.16]	[16.38–64.37]
28 February 2016	VV/VH, 39–40°	[4.48–28.05]	-	[1.28–5]	[28.72–95.10]
15 April 2016	VV/VH, 39–40°	[10.81–23.1]	[0.62–3.24]	[0.03–4.25]	[56.3–112]
9 May 2016	VV/VH, 39–40°	[9.02–23.02]	-	[0.001–0.03]	[76.8–110.7]
23 December 2016	VV/VH, 39–40°	[23.14–41.18]	[0.72–4.55]		-
16 January 2017	VV/VH, 39–40°	[11.96–33.19]	-	[0.05–4.23]	[8.34–25.63]
21 February 2017	VV/VH, 39–40°	[8.97–30.64]	[1.08–3.78]	[0.28–5]	[11.57–62.57]
18 March 2017	VV/VH, 39–40°	[8.12–31.58]	-	[0.58–5]	[18–84.07]
23 April 2017	VV/VH, 39–40°	[6.36–34.46]	-	[0.09–1.6]	[56.93–99.07]

**Table 3:** Calibration parameters and statistical precision coefficients computed with the proposed empirical models, for the VV and VH polarizations, relative mean square error (RMSE) and correlation coefficient R^2^.

Polarisation	*α_pq_*	*β_pq_*	*δ_pq_*	RMSE (dB)	R^2^
pq = VV	0.17	3.25	−15.06	1.47	0.61
pq = VH	0.13	1.88	−23.01	1.29	0.5
